# Does Drinking Location Matter? Profiles of Risky Single-Occasion Drinking by Location and Alcohol-Related Harm among Young Men

**DOI:** 10.3389/fpubh.2014.00064

**Published:** 2014-06-10

**Authors:** Caroline Bähler, Michelle Dey, Petra Dermota, Simon Foster, Gerhard Gmel, Meichun Mohler-Kuo

**Affiliations:** ^1^Institute of Social and Preventive Medicine, University of Zurich, Zurich, Switzerland; ^2^Melbourne School of Population and Global Health, University of Melbourne, Melbourne, VIC, Australia; ^3^Alcohol Treatment Centre, Lausanne University Hospital (CHUV), Lausanne, Switzerland

**Keywords:** drinking locations, drinking profiles, risky single-occasion drinking, negative alcohol-related consequences

## Abstract

In adolescents and young adults, acute consequences like injuries account for a substantial proportion of alcohol-related harm, especially in risky single-occasion (RSO) drinkers. The primary aim of the study was to characterize different drinking profiles in RSO drinkers according to drinking locations and their relationship to negative, alcohol-related consequences. The sample consisted of 2746 young men from the Cohort Study on Substance Use Risk Factors who had reported drinking six or more drinks on a single-occasion at least monthly over the preceding 12 months. Principal component analysis on the frequency and amount of drinking at 11 different locations was conducted, and 2 distinguishable components emerged: a *non-party-dimension* (loading high on theater/cinema, sport clubs, other clubs/societies, restaurants, and sport events) and a *party-dimension* (loading high on someone else’s home, pubs/bars, discos/nightclubs, outdoor public places, special events, and home). Differential impacts of drinking location profiles were observed on severe negative alcohol-related consequences (SAC). Relative to those classified as low or intermediate in both dimensions, no significant difference experiencing SAC was found among those who were classified as high in the *non-party-dimension* only. However, those who were classified as high in the *party-dimension* alone or in both dimensions were more likely to experience SAC. These differential effects remained after adjusting for alcohol consumption (volume and risky single-occasion drinking), personality traits, and peer-influence [adjusted OR = 0.83 (0.68–1.02), 1.57 (1.27–1.96), and 1.72 (1.23–2.41), respectively], indicating independent effects of drinking location on SAC. The inclusion of sociodemographic factors did not alter this association. The fact that this cluster of *party-dimension* locations seems to predispose young men to experiencing SAC has important implications for alcohol control policies.

## Introduction

From the public health perspective, risky single-occasion drinking (RSOD) is considered one of the major problems stemming from alcohol consumption among adolescents and young adults. RSOD has been consistently identified as a stronger predictor of negative alcohol-related consequences among young adults than total drinking volume (1–4). According to the literature, acute consequences like injuries account for a substantial proportion of alcohol-related harm, especially in young people (5–7). In fact, acute consequences of RSOD rank among the main risk factors for mortality and morbidity in late adolescence and early adulthood (7, 8). While the negative consequences of problematic alcohol consumption in young men are well-known, less is known about the relationship between different drinking situations and the consequences of RSOD. Individual factors cannot be the only explanatory variables, as the drinking behavior of the same individual may vary in different settings (9). According to Knibbe et al. (10), exposure to an alcohol-stimulating environment is even more decisive than alcohol-specific beliefs or norms. This might be especially true in a country like Switzerland, where alcohol consumption is integrated into everyday life and where drinking places are multifaceted and likely to influence drinking behavior and its consequences in different ways (1). Previous results have shown that approximately 80% of alcohol consumption does not take place at home or at someone else’s home, but in public places like bars, discos, and festivals (11).

To investigate the relationship between drinking and alcohol-related consequences, not only drinking pattern – including the frequency and volume of drinking over a given time period – but also the demographics (i.e., male gender, age), psychological state (i.e., personality, attitudes), and social context (i.e., peer-influence, drinking locations) of drinkers must be considered (9, 12). A more differentiated understanding of individual and environmental circumstances that contribute to the risk of negative alcohol-related consequences has emerged, revealing risk factors related to the time-frame of drinking and belonging to specific subgroups (13). Given this, considering the differential impact of social embedding of drinking locations on negative alcohol-related consequences could contribute significantly to our understanding of problematic alcohol consumption. However, studies to date have predominantly been conducted in the USA and Canada, and many papers dealing with situational drinking settings have looked specifically at university/campus life (13–16).

The primary aim of the present study was to characterize drinking profiles according to drinking location and their differential impact on negative alcohol-related consequences in a population-based Swiss sample. It can be assumed that specific drinking locations and occasions are associated with a lack of supervision and social control, thereby raising the likelihood of negative consequences. Findings should have further implications for public health interventions aimed at lowering problematic alcohol consumption in young people, which is one of the main strategic goals of the National Alcohol Program 2013–2016 of the Swiss Federal Office of Public Health (17).

## Materials and Methods

All analyses conducted for this study were based on data collected for the Cohort Study on Substance Use Risk Factors (C-SURF). The study design and study sample for C-SURF have been detailed elsewhere (18). The study protocol was approved by the Ethics Committee for Clinical Research at Lausanne University Medical School (Protocol No. 15/07). In brief, participants were enrolled between 23 August 2010 and 15 November 2011 at three out of six Swiss army recruitment centers – in Lausanne (French-speaking), Windisch and Mels (German-speaking) – encompassing 21 of 26 Swiss cantons. As army recruitment is mandatory in Switzerland, all young men of roughly 19 years of age were eligible for study inclusion (i.e., no pre-selection to army conscription exists).

### Participants

Out of the 15,074 young men who showed up at the recruitment centers, 1829 (12.1%) were not seen by the research staff. Among the 13,245 conscripts who were informed about the study, 7563 (57.1%) gave written informed consent to participate, among whom 5990 (79.2%) subsequently filled out the baseline questionnaire. For the purposes of this study, only those men who reported having consumed six or more standard drinks on a single-occasion at least monthly over the preceding 12 months were included in analysis [2746 (45.8%) of the 5990 C-SURF participants], as risky single-occasion (RSO) drinking contributes substantially to the risk of alcohol-related harm in young men. Fifty participants had to be excluded due to missing data on drinking locations. Hence, ultimately, the sample consisted of 2696 subjects.

### Measures

#### Drinking measures

Drinking volume (the average number of drinks per week) was calculated by multiplying the usual frequency (rescaled to days per week) and the usual quantity (number of standard drinks per occasion) of the past 12 months. RSOD was defined in accordance with Murgraff et al. (19), as consuming at least six standard drinks on a single-occasion. This, in turn, was divided into three categories: monthly, weekly, and daily. RSOD referred to the last 12 months, as well. Standard drinks containing 10–12 g of pure alcohol were depicted in the questionnaire.

#### Drinking locations

Drinking locations were categorized according to the 2000 New Zealand National Alcohol Tracking Survey (20). For each location, the usual frequency and usual amount of drinking over the previous 12 months was assessed. Frequency was divided into eight categories, ranging from “never” to “daily” and was rescaled to the number of days per week. The amount per occasion was divided into six categories ranging from “1 to 2 standard drinks per occasion” to “12 or more standard drinks per occasion” and was rescaled to the average number of standard drinks per occasion.

#### Severe negative alcohol-related consequences

Participants were asked whether or not they had experienced negative consequences associated with drinking alcohol over the previous 12 months. To date, no standardized grouping of consequences from drinking exists for epidemiological surveys (21). For our study, the following negative alcohol-related consequences were included, all considered more serious alcohol-related consequences by Wechsler et al. (22): unintended or unprotected sexual intercourse; an accident or injury; conflicts with police/authorities; arguments or fights; property damage. Responses to questions regarding these severe negative alcohol-related consequences (SAC) were dichotomized into “yes” if one or more of the six consequences had occurred or “no.” Extensive analyses of various subgroups of consequences (e.g., looking at sexual or aggressive behaviors separately) failed to reveal any substantial differences in the impact of profile groups by drinking location.

#### Sociodemographic factors

The following sociodemographic factors were assessed by means of self-report: *subject age* (younger than 20 vs. 20 years or older); *type of residence*, defined as “rural” (<10,000 inhabitants) or “urban” (≥10,000 inhabitants); and *linguistic region*, defined as “German” or “French.” Furthermore, data concerning *family affluence* (categorized into “above average income,” “average income,” and “below average income”), the highest achieved *education of the study subject* (categorized into “primary school,” “higher vocational school,” and “high school/university”), and the highest achieved *education of the father* (categorized into “no secondary education,” “secondary education,” and “tertiary education”) were included in analysis.

#### Personality factors

##### Sensation seeking

This personality trait was measured using the Brief Sensation Seeking Scale (BSSS) (23), a scale consisting of eight items, with participants asked to score each statement from 1 (strongly disagree) to 5 (strongly agree).

##### Anxiety/neuroticism, aggression/hostility and sociability

Three different personality traits were measured, in accordance with the Zuckerman–Kuhlmann Personality scale (ZKPQ-50-cc) (24). Each scale consists of 10 items, with subjects asked whether or not they agreed with each of the corresponding statements (0 disagree, 1 agree).

#### Peers

Finally, subjects were asked whether any of their closest friends had what they would call a “significant drinking problem” – meaning one that did or should have led to treatment. Answer categories were as follows: “no one,” “one or two,” “some of them,” and “most of them.”

### Data analysis

Continuous variable data are presented as medians and inter-quartile ranges (IQR), and categorical variable data as percentages. Principle component analysis (PCA) of drinking location was conducted to identify variable combinations. Each of the resulting components was then classified into three categories, according to their factor scores. For this purpose, two cut-off values at the 33.3 and the 66.6 percentiles were applied, and the components divided into low (≤33.3 percentile), intermediate (>33.3 and ≤66.6 percentile), and high (>66.6 percentile), as per their factor scores. Logistic regression models were used to evaluate the associations between SAC and the drinking profiles identified by PCA. SAC served as the dependent variable, and the drinking profiles as independent. Adjustments were made for sociodemographic variables (age, type of residence, linguistic region, family affluence, highest achieved education of the participant, and highest achieved education of the father), personality factors (sensation seeking, anxiety/neuroticism, aggression/hostility, and sociability), and peer-influence in logistic regression models. Classification of cases served to evaluate model adequacy. Statistical analyses were performed using SPSS version 21.0.

## Results

### Characterizing drinking location profiles

To investigate whether drinking locations might cluster in a way as to identify different dimensions of drinking profiles, PCA with varimax rotation was conducted on the frequency and amount of drinking at the 11 different types of location. The factor analysis was exploratory, and two distinguishable components emerged. The two resulting components explained 56.1% of the total variance in the grouping items. The first component was termed as a *non-party-dimension* and included a total of five drinking location items with loadings between 0.65 and 0.86. The second component was termed a *party-dimension*, incorporating six locations with loadings between 0.51 and 0.74 (Table 1). The *non-party-dimension* loaded highly for theater/cinema, sport clubs (e.g., football, hockey, gymnastics), other clubs/societies (orchestra, choir, chess club etc.), restaurants, and sports events. In contrast, the *party-dimension* loaded highly for someone else’s home, pubs/bars, discos/nightclubs, outdoor public places (e.g., parks, swimming pools, streets), special events (e.g., festivals, street parties, carnival, markets, exhibitions, concerts), and at home. Drinking at home was the drinking location with the smallest difference in loading between the two dimensions, meaning that this location contributed the least to any distinction between them.

**Table 1:** **Principal component analysis on drinking locations with varimax-rotated factor solutions (*n* = 2696)**.

Item	Non-party- dimension	Party- dimension
Theater/cinema	0.86	0.12
Sport clubs (e.g., football, hockey, gymnastics)	0.82	0.24
Other clubs/societies (orchestra, choir, chess club etc.)	0.78	0.25
Restaurants	0.70	0.34
Sports events	0.65	0.38
Pubs/bars	0.13	0.74
Someone else’s home	0.25	0.73
Discos/nightclubs	0.21	0.69
Outdoor public places (e.g., parks, swimming pools, streets)	0.25	0.62
Special events (e.g., festivals, street parties, exhibitions)	0.16	0.58
Home	0.30	0.51

### Drinking profiles

The factor scores from the two resulting PCA components were each divided into three categories (low, intermediate, and high). Consequently, subjects were allocated to one of four profile categories: (1) subjects who had low to intermediate factor scores for both the *non-party-dimension* and *party-dimension* (LL, *n* = 1115); (2) those who only had high scores for the *non-party-dimension* (NH, *n* = 683); (3) those who only had high scores for the *party-dimension* (PH, *n* = 682); and (4) those who had high scores for both dimensions (HH, *n* = 216). The mean ages for the four profiles were 19.83 (SD = 1.10), 19.91 (SD = 1.26), 19.98 (SD = 1.10), and 19.88 (SD = 1.09), respectively. Baseline characteristics for these four profiles are shown in Tables 2 and 3.

**Table 2:** **Baseline characteristics of the four drinking profiles^a^ by drinking location**.

Baseline characteristics	LL	NH	PH	HH
*n* (%)	(*n* = 1115)	(*n* = 683)	(*n* = 682)	(*n* = 216)
**RESIDENCE**				
Rural	669 (60.0)	476 (69.7)	425 (62.3)	154 (71.3)
Urban	446 (40.0)	207 (30.3)	257 (37.7)	62 (28.7)
**LINGUISTIC REGION**				
German	526 (47.2)	380 (55.6)	254 (37.2)	103 (47.7)
French	589 (52.8)	303 (44.4)	428 (62.8)	113 (52.3)
**FAMILY AFFLUENCE**				
Above average income	517 (46.4)	333 (48.8)	308 (45.2)	99 (45.8)
Average income	455 (40.8)	259 (37.9)	297 (43.5)	89 (41.2)
Below average income	143 (12.8)	91 (13.3)	77 (11.3)	28 (13.0)
**EDUCATION PARTICIPANT^b^**				
Primary school	581 (52.8)	340 (50.7)	310 (46.5)	95 (44.4)
Higher vocational school	266 (24.1)	208 (31.0)	217 (32.5)	77 (36.0)
High school/university	254 (23.1)	123 (18.3)	140 (21.0)	42 (19.6)
**EDUCATION FATHER^b^**				
No secondary education	89 (8.1)	49 (7.3)	44 (6.5)	15 (7.0)
Secondary education	570 (51.6)	374 (55.4)	370 (54.7)	123 (57.2)
Tertiary education	445 (40.3)	252 (37.3)	263 (38.8)	77 (35.8)

*^a^LL, participants with low to intermediate factor scores for both dimensions; NH, participants with high factor scores for the *non-party-dimension* only; PH, participants with high factor scores for the *party-dimension* only; HH, participants with high factor scores for both dimensions*.

*^b^*N* varied slightly due to missing data*.

**Table 3:** **Personality traits of the four drinking profiles^a^ by drinking location**.

Personality traits	LL	NH	PH	HH
(median; IQR)	(*n* = 1115)	(*n* = 683)	(*n* = 682)	(*n* = 216)
Sensation seeking	3.3 (2.9–3.8)	3.1 (2.6–3.8)	3.6 (3.0–4.0)	3.6 (3.0–4.3)
Anxiety/neuroticism	1 (0–3)	1 (1–3)	1 (0–3)	1 (0–3)
Aggression/hostility	4 (3–6)	4 (3–6)	5 (3–7)	5 (3–7)
Sociability	7 (5–8)	7 (5–8)	7 (5–8)	7 (6–8)

*^a^LL, participants with low to intermediate factor scores for both dimensions; NH, participants with high factor scores for the *non-party-dimension* only; PH, participants with high factor scores for the *party-dimension* only; HH, participants with high factor scores for both dimensions*.

### Severe negative alcohol-related consequences

Almost half (48.9%) of our subjects reported having experienced SAC within the previous 12 months (42.7% among those within the LL profile group, 39.3% among NH, 63.1% among PH, and 66.2% among those within the HH profile group). The most frequently mentioned consequences within the LL profile group were accident or injury (16.8%), unintended sexual intercourse (15.5%), and arguments or fights (14.6%). Corresponding figures were 14.0, 17.8, and 16.3% within the NH profile group, 28.9, 27.5, and 28.2% within the PH profile group, and 31.5, 33.3, and 32.4% within the HH profile group.

Analysis revealed differential impacts of drinking location profiles on SAC among young adults who engaged in RSO within the preceding 12 months. Relative to those classified as low or intermediate for both dimensions, no significant difference in SAC was detected among those classified as high for the *non-party-dimension* alone [crude OR (95% CI) = 0.87 (0.72–1.06)]. However, those classified as high for the *party-dimension* alone or for both dimensions were more likely to report having experienced SAC: crude OR = 2.30 (1.89–2.79) in the PH profile group and 2.63 (1.94–3.57) in the HH profile group.

In order to rule out the possibility that the higher odds of experiencing SAC was solely explained by more drinks being consumed at specific locations, we further adjusted for drinking volume in a regression model. The median (IQR) drinking volumes for the LL, NH, PH, and HH profile groups were 8 (5–12), 8 (5–12), 14 (9–20), and 16 (10–30) standard drinks per week, respectively. Frequency of RSO drinking (monthly vs. weekly vs. daily) also was included in the regression model. After adjusting for drinking volume and RSOD, the OR for SAC was still not significant within the NH profile group [0.83 (0.68–1.00), *p* = 0.055]. In the PH profile group, the adjusted OR fell to 1.79 (1.45–2.20, *p* < 0.001), while the OR in the HH profile group was now 1.88 (1.37–2.60, *p* < 0.001). These results indicate that the effect of drinking location on SAC was only partially mediated through alcohol consumption.

In a second model, the association between the drinking location profiles and SAC was further adjusted for the sociodemographic factors – age, linguistic region, residence, highest achieved education of the participant, family affluence, and highest achieved education of the father (Table 4). However, all the above sociodemographic factors had no influence on the association between drinking profile and SAC. The differences in profile groups relative to SAC remained: OR = 0.83 (0.68–1.01, *p* = 0.062) within the NH profile group relative to LL; OR = 1.79 (1.45–2.21, *p* < 0.001) within the PH profile group; and OR = 1.94 (1.40–2.69, *p* < 0.001) within the HH profile group.

**Table 4:** **Logistic regression models of drinking profiles and sociodemographic variables on negative alcohol-related consequences (*n* = 2620)**.

	OR (95% CI)	*p*-Value
**SEVERE NEGATIVE ALCOHOL-RELATED CONSEQUENCES (SAC)**		
Drinking profiles^a^
LL	1.00	
NH	0.83 (0.68–1.01)	0.062
PH	1.79 (1.45–2.21)	<0.001
HH	1.94 (1.40–2.69)	<0.001
RSOD^b^
Monthly	1.00	
Weekly	1.61 (1.35–1.93)	<0.001
Daily	2.74 (1.54–4.87)	0.001
Drinking volume (standard drinks per week)	1.01 (1.00–1.02)	0.043
Age	1.03 (0.87–1.23)	0.702
Residence
Rural	1.00	
Urban	1.16 (0.98–1.38)	0.087
Linguistic region
German	1.00	
French	1.06 (0.88–1.26)	0.543
Family affluence
Above average income	1.00	
Average income	1.03 (0.80–1.34)	0.810
Below average income	0.93 (0.72–1.20)	0.564
Education participant
Primary school	1.00	
Higher vocational school	1.11 (0.89–1.39)	0.346
High school/university	1.20 (0.94–1.51)	0.140
Education father
No secondary education	1.00	
Secondary education	1.13 (0.81–1.57)	0.472
Tertiary education	0.97 (0.81–1.16)	0.712

*^a^LL, participants with low to intermediate factor scores for both dimensions; NH, participants with high factor scores for the *non-party-dimension* only; PH, participants with high factor scores for the *party-dimension* only; HH, participants with high factor scores for both dimensions*.

*^b^RSOD, risky single-occasion drinking*.

In contrast, when personality factors (sensation seeking, anxiety/neuroticism, aggression/hostility, and sociability) were included in regression analysis, the odds of SAC declined among those within the PH or HH profile group [OR = 1.63 (1.32–2.02), *p* < 0.001, and OR = 1.76 (1.26–2.46), *p* = 0.001, respectively], and remained unchanged within the NH profile group [OR = 0.83 (0.68–1.01), *p* = 0.062]. All the above personality traits – except for sociability – were significantly associated with an increased likelihood of SAC. However, personality traits failed to fully explain differences in the association between drinking profiles and acute negative alcohol-related consequences.

In a fourth and final model, the influence of peers with significant drinking problems was added to personality variables. This adjustment exerted a small influence on the association between drinking profiles and SAC. Again, the OR of the NH profile group did not change [OR = 0.83 (0.68–1.02), *p* = 0.075], whereas the OR of SAC slightly decreased in the PH and HH profile groups [OR = 1.57 (1.27–1.96), *p* < 0.001, and OR = 1.72 (1.23–2.41), *p* = 0.001, respectively] (Table 5). Approximately two-thirds (64.0%) of subjects were correctly classified as having experienced negative alcohol-related consequences in this final model.

**Table 5:** **Logistic regression models of drinking profiles, personality traits and peers on negative alcohol-related consequences (*n* = 2668)**.

	OR (95% CI)	*p*-Value
**SEVERE NEGATIVE ALCOHOL-RELATED CONSEQUENCES (SAC)**		
Drinking profiles^a^
LL	1.00	
NH	0.83 (0.68–1.02)	0.075
PH	1.57 (1.27–1.96)	<0.001
HH	1.72 (1.23–2.41)	0.001
RSOD^b^
Monthly	1.00	
Weekly	1.42 (1.18–1.70)	<0.001
Daily	2.36 (1.31–4.26)	0.004
Drinking volume (standard drinks per week)	1.01 (1.00–1.01)	0.212
Personality traits
Sensation seeking	1.51 (1.36–1.69)	<0.001
Anxiety/neuroticism	1.08 (1.04–1.13)	<0.001
Aggression/hostility	1.16 (1.12–1.21)	<0.001
Sociability	1.03 (0.99–1.08)	0.140
Peers with significant drinking problems
None	1.00	
One or two	1.59 (0.91–2.78)	0.104
Some of them	2.16 (1.52–3.05)	<0.001
Most of them	1.37 (1.12–1.66)	0.002

*^a^ LL, participants with low to intermediate factor scores for both dimensions; NH, participants with high factor scores for the *non-party-dimension* only; PH, participants with high factor scores for the *party-dimension* only; HH, participants with high factor scores for both dimensions*.

*^b^ RSOD, risky single-occasion drinking*.

## Discussion

Principle component analysis on the frequency and amount of drinking at 11 different types of drinking location revealed two dimensions: a *non-party-dimension* that loaded highly for theater/cinema, sport clubs, other clubs/societies, restaurants, and sport events; and a *party-dimension* that loaded highly for drinking at someone else’s home, pubs/bars, discos/nightclubs, outdoor public places, special events, and drinking at home.

Subsequent regression analysis revealed differential impacts of drinking location profiles on SAC among adolescents and young adults who had engaged in RSO drinking over the previous 12 months. Relative to those classified as low or intermediate for both dimensions, no significant difference in reported SAC was found among those classified as high for the *non-party-dimension* only. However, those who were classified as high for the *party-dimension* alone or for both dimensions were more likely to experience SAC. These effects persisted after adjusting for a variety of variables, including the degree of alcohol consumption, indicating that drinking location has independent effects on drinking consequences.

Differences in the associations between the drinking profiles and alcohol-related problems were attenuated, but persisted after controlling for alcohol consumption (volume and RSOD), personality traits, and peer-influence, indicating independent effects of these variables on SAC. In contrast, including sociodemographic factors did not alter the associations. The self-selection hypothesis of choosing drinking locations based on personality traits (9) only explained some of the variability in drinking patterns and alcohol-related consequences. Therefore, the cluster of locations that had high factor scores for the *party-dimension* seemed to predispose young men to experiencing SAC.

Location-specific differences related to RSOD and drinking problems have been examined in several previously published studies. In a study by Single and Wortley (25), drinking in bars and at parties was more strongly associated with self-reported drinking problems over the past 12 months than drinking in restaurants, especially among young men. In that study, the strength of the association between drinking in bars and experiencing problems was attenuated, but still remained significant, after controlling for the level of consumption. As in our study, education was no longer significantly associated with drinking problems, once the relationship was controlled for drinking volume and RSOD (25).

Our results are also partly consistent with those of Stockwell et al. (26), who demonstrated that drinking in nightclubs is associated more strongly with alcohol-related harm than drinking in restaurants. A higher likelihood of negative drinking consequences during event-specific occasions like festivals was also reported in the latest review by Mallett et al. (13). However, to the best of our knowledge, none of the previous studies clustered drinking locations or assessed the association of these clusters on negative alcohol-related consequences.

Sociodemographic variables could not explain the difference in the association between drinking profile by drinking location and SAC. One reason could be that only young males were included in our study. Furthermore, our results are consistent with previous studies that failed to identify socio-economic status or age as independent predictors of alcohol-related consequences once drinking patterns were controlled for (27, 28). In addition, research has shown that peers may influence the association between drinking patterns and negative alcohol-related consequences (29). In the present analysis, the association was modified by the inclusion of the number of peers with significant drinking problems, though differences in the relationship persisted. However, the peer measure included in this model did not specifically look at RSOD, but rather at problematic alcohol consumption as a whole. A more differentiated peer variable might have had an even greater impact upon our results. As drinking is more often related to social interactions as many other health-relevant behaviors, preventive measures should take drinking situations, as expressions of different social interactions, into account (3).

### Limitations

Our study has several potential limitations. First, all data were self-reported. Results may therefore be distorted because of assessment bias, recall bias, or social desirability. For example, students willing to experience consequences might have been more likely to report such consequences (13). Moreover, cultural differences attributing consequences to alcohol have been observed by others (21). Furthermore, expectations regarding the effect of alcohol consumption could have influenced the relationship between drinking profile and alcohol-related harm (30), it is conceivable that further factors might have influenced our study findings, such as family history or drinking companions. However, in the study by Wells et al. (31), drinking companions did not confound the relationship between alcohol consumption and alcohol-related aggression. Likewise, “pre-drinking” or “pre-gaming” could have confounded the association between drinking profile by drinking location and SAC (13, 14), even though we indirectly controlled for this behavior by including drinking volume. The comprehensive C-SURF questionnaire allowed us to control for a variety of sociodemographic and other individual variables. In addition, we did not control for drug use in our final model because the results did hardly change after including drug use. Moreover, drug use is illegal in Switzerland. Therefore, it is unlikely that the young men would use drugs in public places like restaurants. Thirdly, the study is subject to selection bias, in that all analyses were limited to young men. The results can therefore not be generalized to women or to older populations. Moreover, the relatively low response rate we observed creates the risk of selection bias. However, because we also obtained short screening questionnaires from 94% of all conscripts at the recruitment centers, we were able to analyze potential non-participation biases by different types of substance use, due to the need for informed consent. In addition, the differences between responders and non-responders among consenters were analyzed. Although some statistically significant differences were noted, they were small in magnitude, and their statistical significance largely due to the large sample. Even though consenters included more drinkers than non-consenters did, substance users were less likely to respond than non-users among consenters. These results have been published in detail elsewhere (18). Finally, the cross-sectional design does not allow for any causal conclusions to be drawn.

### Implications

Numerous studies have demonstrated that problematic alcohol consumption, like RSO drinking, is widespread in young people (32, 33); and that such consumption is associated with negative consequences (2, 3, 5). The results of our study suggest that drinking location might have an important influence on alcohol-related harm above and beyond alcohol consumption *per se*. We discovered a cluster of drinking locations that are not bound to a specific type of drinker, like sensation seekers, indicating independent effects of drinking location on SAC. This, in turn, has important implications for alcohol control policies. It supports the need for structured measures like policies, regulations, and compliance checks tailoring this cluster of drinking locations to substantially reduce alcohol-related harm.

On one hand, compliance with alcohol policies and existing legislation at sensible locations with on-premise alcohol purchases – like pubs/bars, discos/nightclubs, and special events – should be monitored more closely. For instance, continuing to serve obviously intoxicated customers has been shown to be the best predictor of negative alcohol-related consequences (26). However, studies conducted in Switzerland and the US have also revealed that compliance with legislation is far from satisfactory (34, 35). A further step could be the adoption of a designated drivers program to reduce drinking and driving (36). The recent introduction of graduated driver licensing in Switzerland will hopefully further reduce automobile collisions among young drivers (37). Additional statutory regulations are needed, such as claims for additional municipal charges for alcoholic beverages at special events (38). On the other hand, preventive measures are needed for off-premise alcohol sellers to reduce alcohol-related harm at locations like outdoor public places and at someone else’s home. Such preventive measures should include restrictions related to outlet liability, alcohol price discounts, and selling hours (39). Moreover, as 89.9% of the participants still lived in their parents’ home, parents also need to be sensitized to the cluster of places associated with alcohol-related harm. Increased parental monitoring and/or regulation might help to reduce negative consequences. In addition, although some of the Swiss campaigns and prevention programs have been designed to be location-specific – like “Safer Dance Swiss” referring to festivals, and “Safer Clubbing” referring to discos/nightclubs – the current results imply that interventions that target single drinking locations only will fall short of preventing SAC in RSO drinkers. The different interventions need to work hand in hand to reduce the burden of SAC. Furthermore, innovations are needed to protect people from the consequences of their binge-drinking behaviors, like free ride programs to ensure that drinkers travel home safely, analogous to proposed student volunteer escort services that assist students walking to their dorms (15).

The fact that negative alcohol-related consequences from RSOD may yet appear in the early adulthood emphasizes the need for timely preventive measures. Successful interventions in early life may not only reduce negative alcohol-related consequences during adolescence, but also prevent adult drinking problems (29).

In summary, it can be stated that a structure of timely preventive measures is needed to reduce alcohol-related harm. However, as the availability of alcoholic beverages is high and there is a long tradition of including alcohol consumption in daily life in Switzerland, the implementation of structural changes poses several challenges (38), something that was illustrated impressively during the latest parliamentary debate in the fall of 2013.

## Conclusion

The present findings suggest that problematic alcohol consumption and negative alcohol-related consequences are strongly associated with a cluster of specific drinking locations. This may, together with further, recently collected data, serve as a basis for specific targeting of public health interventions that aim to lower risky drinking patterns and their negative consequences.

Since RSO drinkers with different cultural backgrounds or different demographics, like higher age or female gender, may select different drinking locations, research also is needed to positively influence regulations drafted by policy makers (21, 28, 40, 41). Future research should also include additional variables related to drinking location, like drinking companions or drinking motives (42, 43).

## Author Contributions

Gerhard Gmel and Meichun Mohler-Kuo are the study’s main investigators. Caroline Bähler and Meichun Mohler-Kuo conceptualized the manuscript. Caroline Bähler analyzed the data and wrote the first draft of this manuscript. Michelle Dey and Simon Foster assisted in data analyses. Gerhard Gmel made major contributions to the content of the manuscript. Petra Dermota was involved in data collection and questionnaire development. All authors contributed to manuscript writing and helped to improve it.

## Conflict of Interest Statement

The authors declare that the research was conducted in the absence of any commercial or financial relationships that could be construed as a potential conflict of interest.
